# Essential Information About Chronobiology and Chronotherapy for the Optimal Care of People With Bipolar Disorders: An Expert Consensus

**DOI:** 10.1111/bdi.70141

**Published:** 2026-07-10

**Authors:** Jacob J. Crouse, Victoria Loblay, Anthony Jorm, Timothy R. Wong, Zsofi de Haan, Carla Gorban, Mirim Shin, Emiliana Tonini, Chris Aiken, Lauren B. Alloy, Kürşat Altinbaş, Serge Beaulieu, Joanne S. Carpenter, Bruno Etain, Yuichi Esaki, Jess G. Fiedorowicz, Corrado Garbazza, Pierre A. Geoffroy, Bartholomeus C. M. Haarman, Tone E. G. Henriksen, Maria Paz Hidalgo, Maree L. Inder, Raymond W. Lam, Helle Ø. Madsen, Colleen A. McClung, Gunnar Morken, Laura Palagini, James Phelps, Richard J. Porter, Aswin Ratheesh, Rixt F. Riemersma‐Van der Lek, Janusz K. Rybakowski, Erika F. H. Saunders, Peter F. J. Schulte, Daniel J. Smith, Holly A. Swartz, Bryan K. Tolliver, Anna Wirz‐Justice, Ian B. Hickie, John F. Gottlieb, the Chronobiology & Chronotherapy Task Force of the International Society for Bipolar Disorders

**Affiliations:** ^1^ Brain and Mind Centre The University of Sydney Sydney New South Wales Australia; ^2^ Centre for Mental Health and Community Wellbeing, Melbourne School of Population and Global Health The University of Melbourne Melbourne Victoria Australia; ^3^ NYU Lagone School of Medicine New York New York USA; ^4^ Department of Psychology and Neuroscience Temple University Philadelphia Pennsylvania USA; ^5^ Department of Psychiatry, Faculty of Medicine Atlas University Istanbul Türkiye; ^6^ Department of Psychiatry, Faculty of Medicine McGill University Montreal Québec Canada; ^7^ Université Paris Cité Paris France; ^8^ Department of Psychiatry Fujita Health University School of Medicine Toyoake Japan; ^9^ The Ottawa Hospital, Ottawa Hospital Research Institute, and University of Ottawa Ottawa Ontario Canada; ^10^ Centre for Chronobiology University of Basel Basel Switzerland; ^11^ Centre ChronoS, GHU Paris—Psychiatry & Neurosciences Paris France; ^12^ University Medical Center Groningen Groningen the Netherlands; ^13^ Division of Mental Health Care Valen Hospital Valen Norway; ^14^ Department of Research and Innovation Fonna Health Trust Haugesund Norway; ^15^ Universidade Federal do Rio Grande do Sul (UFRGS) Porto Alegre RS Brazil; ^16^ Department of Psychological Medicine University of Otago Christchurch New Zealand; ^17^ Department of Psychiatry University of British Columbia Vancouver British Columbia Canada; ^18^ Copenhagen Affective Disorder Research Center (CADIC), Mental Health Center Copenhagen Copenhagen Denmark; ^19^ Department of Psychiatry, Translational Neuroscience Program University of Pittsburgh School of Medicine Pittsburgh Pennsylvania USA; ^20^ Department of Mental Health St Olav University Hospital Trondheim Norway; ^21^ Faculty of Medicine NTNU: Norwegian University of Science and Technology Norway; ^22^ Department of Neuroscience, Psychiatric Section University of Pisa Pisa Italy; ^23^ Medical Director, DepressionEducation.org USA; ^24^ Discipline of Psychiatry and Mental Health, School of Clinical Medicine University of New South Wales Randwick Australia; ^25^ Mental Health Services Drenthe Assen the Netherlands; ^26^ Department of Adult Psychiatry Poznan University of Medical Sciences Poznan Poland; ^27^ Department of Psychiatry and Behavioral Health Pennsylvania State University College of Medicine Hershey Pennsylvania USA; ^28^ Mental Health Services Noord‐Holland‐Noord Alkmaar the Netherlands; ^29^ Division of Psychiatry, Institute for Neuroscience and Cardiovascular Research University of Edinburgh Edinburgh UK; ^30^ University of Pittsburgh School of Medicine Pittsburgh Pennsylvania USA; ^31^ Addiction Sciences Division, Department of Psychiatry and Behavioral Sciences Medical University of South Carolina Charleston South Carolina USA; ^32^ Chicago Psychiatry Associates Chicago Illinois USA

**Keywords:** bipolar disorder, chronotherapeutic, circadian, mood, pathophysiology, rhythms, treatment

## Abstract

**Introduction:**

Circadian dysfunction is involved in the pathophysiology of bipolar disorders (BD), and circadian‐based interventions are gaining recognition in their management. Moreover, basic and epidemiologic research has generated findings inspiring circadian‐informed self‐ and clinician‐management strategies. Despite these gains, many Clinical Practice Guidelines and clinical training programs have not incorporated this evidence in their recommendations and curricula. This International Society for Bipolar Disorders (ISBD) Chronobiology and Chronotherapy Task Force position paper reports a Delphi‐based expert consensus on what is essential for mental health clinicians to know about the chronobiology and chronotherapy of BD.

**Methods:**

An initial pool of statements was extracted from academic and grey literature, and experts could suggest additional statements. Statements were rated on a 5‐point scale (‘essential’; ‘important’; ‘don't know/depends’; ‘unimportant’; ‘should not be included’). Consensus was reached when statements were rated as essential or important by ≥ 80% of experts.

**Results:**

Thirty experts from 15 countries in Europe, North and South America, and the Asia Pacific participated (mean age of 55.3 years [SD = 11.8]; 40% female; 83% psychiatrists; mean clinical experience of 26 years [SD = 10.8]). Eight‐hundred‐and‐thirty‐seven statements were rated across three rounds. Consensus was reached on 342 statements spanning four major themes: basic circadian science; circadian health and disruption; chronobiology of BD; and six chronotherapies (e.g., protocols, outcomes, risks/contraindications).

**Conclusions:**

An expert consensus was obtained on the essential information about the chronobiology and chronotherapy of BD, intended to help clinicians optimise their management of BD. Dissemination of this knowledge is expected to enhance the training and efficacy of clinicians.

## 1 Introduction

There are several major models of the pathophysiology of bipolar disorders (BD) that inform their diagnosis and treatment. These models include disturbances in reward function, mitochondrial bioenergetics, monoamine signalling, and neurotrophic and neuroinflammatory processes [1, 2, 3]. Over the past 50 years, the basic science of chronobiology, and the applied field of chronotherapeutics, have generated another foundational model for understanding and treating BD [1, 2, 4, 5]. In the 1970s–1980s, landmark studies emerged suggesting that disturbances in circadian rhythms contribute to the pathophysiology of BD [6, 7, 8, 9, 10]. Supportive evidence has since accumulated across many approaches including genetic [4], family [11, 12], epidemiologic [13], longitudinal [14, 15], physiologic [4, 16, 17, 18, 19, 20], biobehavioural [4, 14, 21, 22, 23], and therapeutic [24]. The hypothesis of a core role of circadian rhythms in BD is now more widely accepted [4].

### 1.1 Gaps in Guidelines

Despite these gains, dissemination of information to clinicians about the chronobiology and chronotherapeutics of BD remains limited. This gap is clearest for Clinical Practice Guidelines (CPGs). A review of 25 CPGs for BD found that fewer than half included even a basic discussion of circadian disturbances [25]. The authors concluded, ‘Given the evidence regarding the importance of circadian dysrhythmias in the onset, course and prognosis of BD, we suggest that omission of information and practical advice on management needs addressing in future updates of CPGs for BD’. The review [25] highlighted the contrast between the promising data about chronotherapeutics for BD [24] and the limited discussion in CPGs of circadian dysfunction both as a pathophysiology and treatment target. For example, in the case of Bright Light Therapy there are now at least eight randomised controlled trials (RCTs) suggesting its efficacy, safety, and tolerability in the treatment of bipolar depression [26, 27], and there is now RCT evidence for Interpersonal and Social Rhythm Therapy [28, 29] and dark therapy using blue‐blocking glasses [30]. Some CPGs do indeed give strong attention to chronobiology regarding the aetiology and pathophysiology of BD (e.g., 2020 Royal Australian and New Zealand College of Psychiatrists CPG [31]) whereas others do not. For example, the words ‘circadian’, ‘chronobiology’, and ‘chronotherapy’ do not appear in the 2018 CANMAT/ISBD CPG [32] (although Bright Light Therapy is discussed regarding bipolar depression).

In addition to RCT evidence, a wealth of clinically‐informative findings comes from observational studies which encourage circadian‐based self‐management strategies (e.g., behavioural regulation of the timing of sleep, wake, and light exposure). Many observations from naturalistic and cohort studies that are missing from, or minimised in, CPGs can be used to help clinicians manage and anticipate their patients' course of illness. For instance, knowing a patient's chronotype can inform prognosis and treatment, as higher morningness chronotypes tend to respond better to lithium, whereas higher eveningness chronotypes have lower success with such treatments, and are more likely to present with challenging features like suicidality [33]. A second example is the knowledge that dampened circadian rhythms are often a core feature of BD, which may prompt greater inclusion of ‘rhythm‐boosting’ strategies into treatment plans. A third example is in the identification of a seasonal pattern of illness, which can help clinicians plan the timing and intensity of strategies and treatments during destabilising periods. There are many other examples related to time‐zone travel, exposure to artificial light at night, and shift work, among others, for which observational evidence may fill in the gaps in which RCTs have not been conducted.

### 1.2 Gaps in Training

A second area in which information about the chronobiology and chronotherapy of BD appears to have been neglected is in clinical training programs. First, a survey of 409 medical schools in 12 countries reported that the average amount of time spent on sleep education is less than 3‐h, with around one‐quarter of respondents suggesting a total absence of sleep curricula [34]. Our assumption is that education about chronobiology is likely to be covered even less than sleep, given that the area of chronobiology has historically received less attention than sleep in the domains of public interest [35], research (i.e., ~320,000 PubMed articles on ‘sleep’ and ~115,000 on ‘circadian’ or ‘chronobiology’), and clinical uptake [36, 37], in addition to circadian rhythms often being incorrectly conflated with sleep [38]. A second survey of 39 chief residents from North American psychiatry programs reported that only about one‐third of programs offered clinical sleep medicine rotations, with some of these programs covering the topics of ‘circadian rhythm disorders’ and ‘psychiatric disorders and sleep’, although it is not clear what the actual rates were [39]. A third survey, which to our knowledge is the only publication investigating education about chronobiology specifically, reported that 76% of surveyed psychologists in Brazil had no academic exposure to biological rhythms, 67% were unfamiliar with the term ‘chronobiology’ (which was coined 50+ years ago), and 63% could not describe a biological rhythm beyond the sleep–wake cycle [40] (which is a hybrid rhythm jointly determined by circadian and sleep homeostatic processes). In Box 1, we supplement this published evidence with an anecdote from a Lived Experience Researcher, who was involved in this study, who recounts their lack of exposure to information about chronobiology and chronotherapy in their encounters with medical practitioners. In the face of this general neglect, the major professional organisation dedicated to chronobiology, the *Society for Research on Biological Rhythms*, recently endorsed the need for a ‘Circadian Medicine Course’ across health and medical fields [41]. We argue that there may be value in BD‐specific curricula.

BOX 1 Perspective by a lived experience researcher with bipolar disorder.*

I would count myself one of the lucky ones. As soon as I was diagnosed with bipolar disorder, I received the best available care. This was because it was informed by the evidence that circadian rhythms play a key role in bipolar, which is essential to understand if you are going to treat the condition effectively. From my treating specialist, I learnt how I could drive my own care by engaging in circadian‐based interventions such as waking up at the same time every morning and taking walks in the morning sunlight. I came to understand how these self‐initiated strategies could regulate my mood and improve my sleep. Knowing that bipolar symptoms can be exacerbated by seasonal changes in light, my specialist and I can closely monitor my symptoms during these periods and draw on chronotherapy, not just as a treatment for symptoms, but as a protective intervention. Understanding that bipolar is linked to physiological changes in circadian rhythms within my body, I can also comprehend that my mental health condition is not who I am, but a physical illness that can benefit from medical treatment and self‐management, no different from any other complex physical illness, such as diabetes or hypertension.

Sadly, while my first symptoms of bipolar disorder occurred when I was just 14 years old, I did not receive the accurate diagnosis of bipolar disorder and access to this appropriate chronobiology‐informed treatment until I was 25. This caused great devastation in my life and unnecessarily prolonged my suffering. I have often wondered whether this subsequent delay in appropriate treatment may have been avoided had the health professionals I interacted with along my journey understood the role of circadian rhythms in bipolar and how this presents.

It may come as a surprise that I was not diagnosed until I was 25 years old, given that I saw my first psychiatrist when I was just 14 and continued to engage with psychiatrists regularly from that point in time. I presented as a young adolescent to both general practitioners and psychiatrists with what I now know to be classic circadian depression symptoms associated with bipolar, including crippling fatigue, impaired sleep quality and concentration, and psychomotor retardation, among others. If these symptoms had been assessed through a chronobiology lens, I find it difficult to believe that I would not have received a proper diagnosis and optimised treatment at an earlier stage in my life. This may have spared me and my loved ones a great deal of pain, allowed me to learn how to self‐manage my condition through psychoeducation of circadian‐based strategies, and reduced self‐stigma and the stigma of others.

The health professionals I have interacted with across my life have been well‐intentioned but provided under‐informed care. While they have sought to offer compassionate care, it has been hindered by profound knowledge gaps comprised of a lack of understanding of the role of chronobiology in bipolar disorder. I believe an attuned health professional with knowledge of the biology of bipolar would have been able to easily assess and identify my condition a mile away, provided appropriate chronotherapeutic interventions, and empowered me to drive my own care. Had this taken place when I first started experiencing my symptoms, this would have been life changing, in the same way I have flourished since my care began being informed by chronobiology in my mid‐twenties. For the first time since I got sick, I feel like me again.

*This person has clinical and research training and works for/with the team in a salaried capacity.

### 1.3 Current Study

The science of chronobiology and chronotherapeutics in BD is complex. There is no consensus to guide what information should be disseminated to help clinicians optimise their care of people with BD. While clinicians' guides (e.g., [42, 43]) and many online articles (of highly variable quality) summarise strategies to manage sleep–wake cycles and circadian rhythms in BD, the amount of information in this area is vast [4].

Accordingly, this *International Society for Bipolar Disorders* (ISBD) *Chronobiology and Chronotherapy Task Force* position paper reports an expert consensus (created via a Delphi methodology) on the essential information for clinicians supporting people with BD to know about the chronobiology and chronotherapy of BD to optimise their care.

## 2 Materials and Methods

### 2.1 Ethical Approval

The study was approved by the University of Sydney's Human Research Ethics Committee (2023/981). Prospective participants were given a Participant Information Sheet and told that advancing through the questionnaire implied consent to participate.

### 2.2 Study Design

The study followed the key elements of the Delphi method [44]. First, the facilitators designed and organised the Delphi study. They compiled a questionnaire with a list of statements about BD and chronobiology or chronotherapy for experts to rate for agreement. A statistical criterion was chosen to operationalise consensus among the aggregated expert ratings. Experts could suggest new statements in the initial round for rating in subsequent rounds. The facilitators shared anonymous feedback to the experts about how their ratings compared to the rest of the panel. Experts could then revise their ratings after receiving feedback. Ratings were aggregated across rounds, with the statistical criterion being used to operationalise consensus and to determine which statements needed re‐rating in a later round.

### 2.3 Creation of the Initial Statements (Round 1)

The facilitators extracted statements for Round 1 of the Delphi study from three sources: (1) reviews published by the ISBD *Chronobiology and Chronotherapy Task Force* [4, 24, 45]; (2) a systematic search of primary studies, systematic reviews, and meta‐analyses on BD and chronobiology or chronotherapy published after 2019 (after the first ISBD *Chronobiology and Chronotherapy Task Force* review); and (3) a systematic search of grey literature on the topic in the US, UK, and Australia (*n* = 1126 unique records; see Supporting Information for a description of the search methodology).

There was no a priori limit on the number of statements to be extracted. In total, 960 statements were extracted and refined by the facilitators, which included rephrasing for clarity, synthesising conceptually similar statements, or deleting those out of scope. This resulted in 758 statements eligible for rating in Round 1.

### 2.4 Recruitment of Experts

The facilitators contacted representatives from three major professional organisations in the topic area: ISBD, Society for Light Treatment and Biological Rhythms (since renamed the Society for Light, Rhythms, and Circadian Health), and the Center for Environmental Therapeutics. The representatives sent an email invitation to their memberships, and recipients were asked to send it to relevant colleagues (i.e., snowball sampling). Participating experts were guaranteed co‐authorship on the study publication if they completed all rounds (a priori maximum = 3) of the Delphi study.

There is no formal sample size calculation for Delphi studies [46, 47]. Publications suggest that the results of single stakeholder Delphi studies stabilise [44], and achieve moderate replicability, with 20–30 participants [46]. Therefore, a required sample size of 30 experts was set for Round 1.

### 2.5 Questionnaire

The questionnaire was deployed in REDCap. The study landing page included the Participant Information Sheet and an eligibility screener, with inclusion criteria being: (i) aged ≥ 18; and (ii) clinician with ≥ 10 years treating BD; or (iii) researcher with ≥ 3 first author papers or ≥ 5 total papers in BD and chronobiology/chronotherapy.

Experts were presented with instructions on how to rate the statements. They were told that their responses were anonymous and were asked, for each statement, to consider the question: ‘How important is it that a clinician treating people with bipolar disorder knows this information?’ Statements were rated on a 5‐item Likert scale: Essential; Important; Don't know/Depends; Unimportant; and Should not be included.

There is no gold standard for defining consensus in a Delphi study [44, 47]. Before conducting the study, we chose a threshold that required ≥ 80% of experts to endorse a statement as *Essential* or *Important*, on the basis of ≥ 80% being a commonly employed threshold in mental health research [47, 48, 49], and to be consistent with Delphi studies conducted by three independent ISBD Task Forces [50, 51, 52]. Statements rated by < 70% as *Essential* or *Important* were discarded. Statements rated by 70%–79% as *Essential* or *Important* were re‐rated in a later round. (Note: the *Don't know/Depends* ratings were included in the denominator when calculating percentages, such that the ≥ 80% threshold refers to all experts, not just experts with a definitive opinion.) For ease of comprehension, the statements were presented within 12 ‘core constructs’: (i) Basics of the mammalian circadian system; (ii) Circadian health and disruption; (iii) Assessing circadian rhythms in clinical practice; (iv) The chronobiology of BD; (v) Chronotherapy: General concepts; (vi) Sleep and circadian hygiene; (vii) Bright light therapy; (viii) Wake therapy; (ix) Dark therapy; (x) Interpersonal and Social Rhythm Therapy; (xi) Melatonin and melatonergic agonists; and (xii) Cognitive Behaviour Therapy for Insomnia adapted for BD.

Round 2 included statements from Round 1 needing re‐rating or rephrasing for clarity/accuracy, as well as new statements suggested by the experts in Round 1. A visual summary (histogram) of the ratings for the ‘borderline’ statements from Round 1 (those rated as *essential* or *important* by 70%–79%) was presented, and experts could choose to retain their original rating or change it. Finally, Round 3 only included statements from Round 2 requiring re‐rating.

### 2.6 Review for Acceptability

In an effort to reduce use of stigmatising language, two ‘lived experience researchers’ [53] who live with BD and are employed in academic research in mood disorders were involved in the study design and reviewed the statements from Round 1 (~50% each). They provided feedback with attention to bias, language, and appropriateness.

### 2.7 Statistical Analysis

**FIGURE 1:**
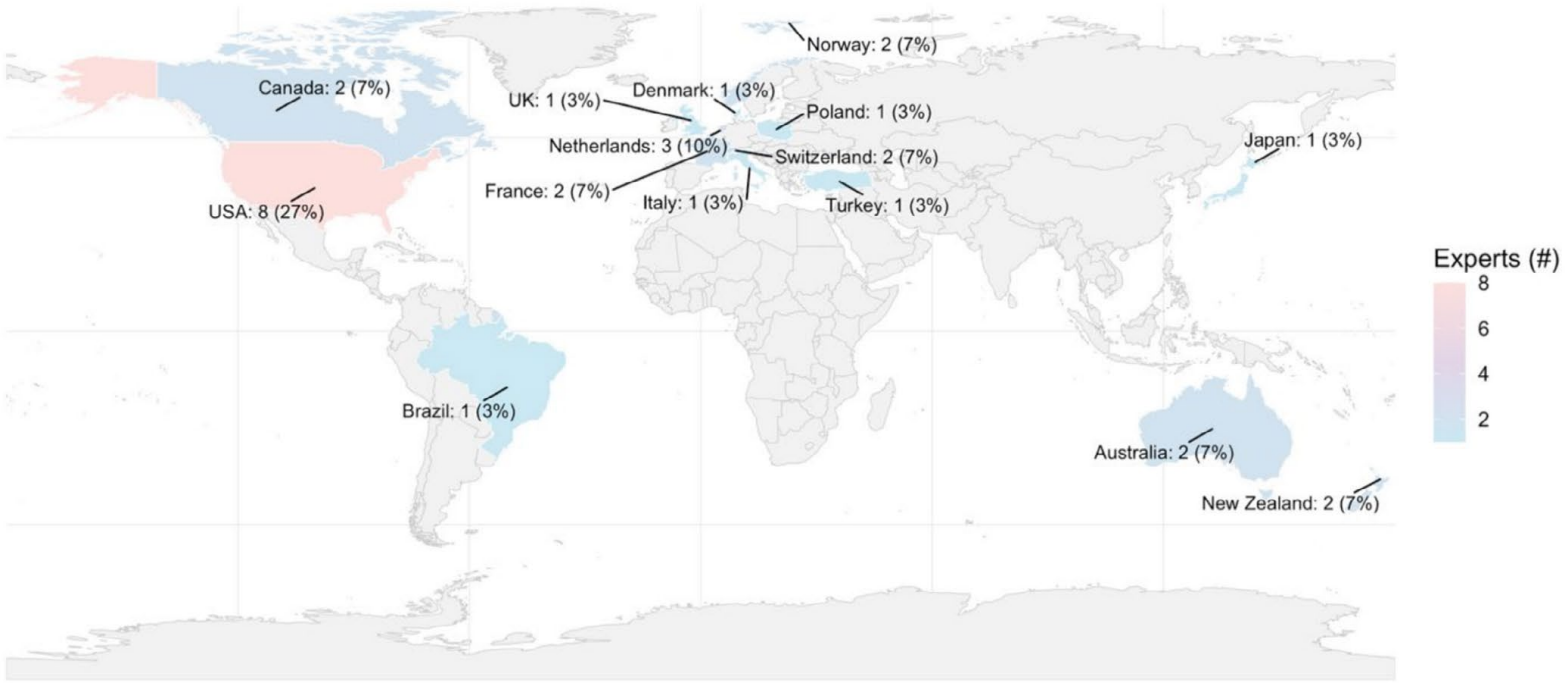
Map of experts (*n* = 30). Based on responses to, “In what country have you principally had experience with bipolar disorders research or treatment?”.

Analyses were conducted using the R programming language in RStudio (4.3.3) [54]. Standard descriptive analyses were undertaken to describe the expert sample and the ‘rnaturalearth’ [55] R package was used to create a world map of the experts (Figure 1). For each item being considered in the Delphi survey by the experts, we calculated the proportion (%) of experts that rated any of the five Likert response categories, and then used the pre‐defined cut‐offs to decide whether each item was discarded (< 70% rating the item as *essential* or *important*), re‐rated (70%–70% rating as *essential* or *important*), or had consensus (80%–100% rating as *essential* or *important*).

## 3 Results

### 3.1 Expert Consensus Panel

The characteristics of the 30 experts who completed the Delphi study (100% of whom were retained from Round 1) are summarised in Table 1. The mean age was 55.3 years (SD = 11.8); 40% were female. The sample was enriched with strong domain expertise, including ~26 years (SD = 10.8) of clinical experience and ~22 years (SD = 12.6) of research experience. The majority were psychiatrists (83%). Most of the experts identified as White (87%), but there was representation from 15 countries in Asia, Europe, North and South America, and Oceania (Figure 1).

**TABLE 1:** Characteristics of the experts (*N* = 30).

Characteristic	Mean (SD) or *N* (%)
Age (years)	55.3 (11.8)
Gender	
Female	12 (40)
Male	18 (60)
Ethnicity	
Asian	3 (10)
Hispanic	1 (3)
White	26 (87)
Experience (years)	
Research	22.3 (12.6)
Clinical	26.0 (10.8)
Researcher type	
Primarily applied	21 (70)
Combination applied/basic	9 (30)
Clinician type	
Social worker	1 (3)
Psychiatrist	25 (83)
Not a clinician	4 (13)

#### 3.1.1 Expert Consensus Statements

**FIGURE 2:**
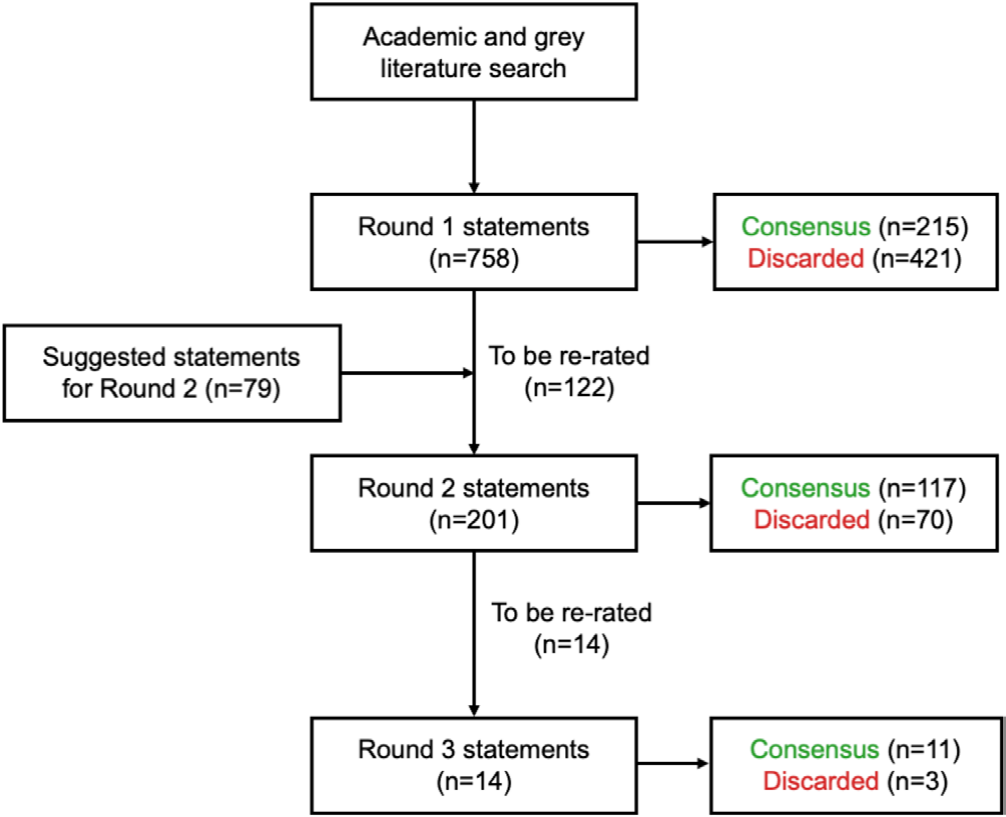
Flow of statements across the Delphi study (*n* = 30 experts). A total of 342 statements reached consensus after all three rounds.

**FIGURE 3:**
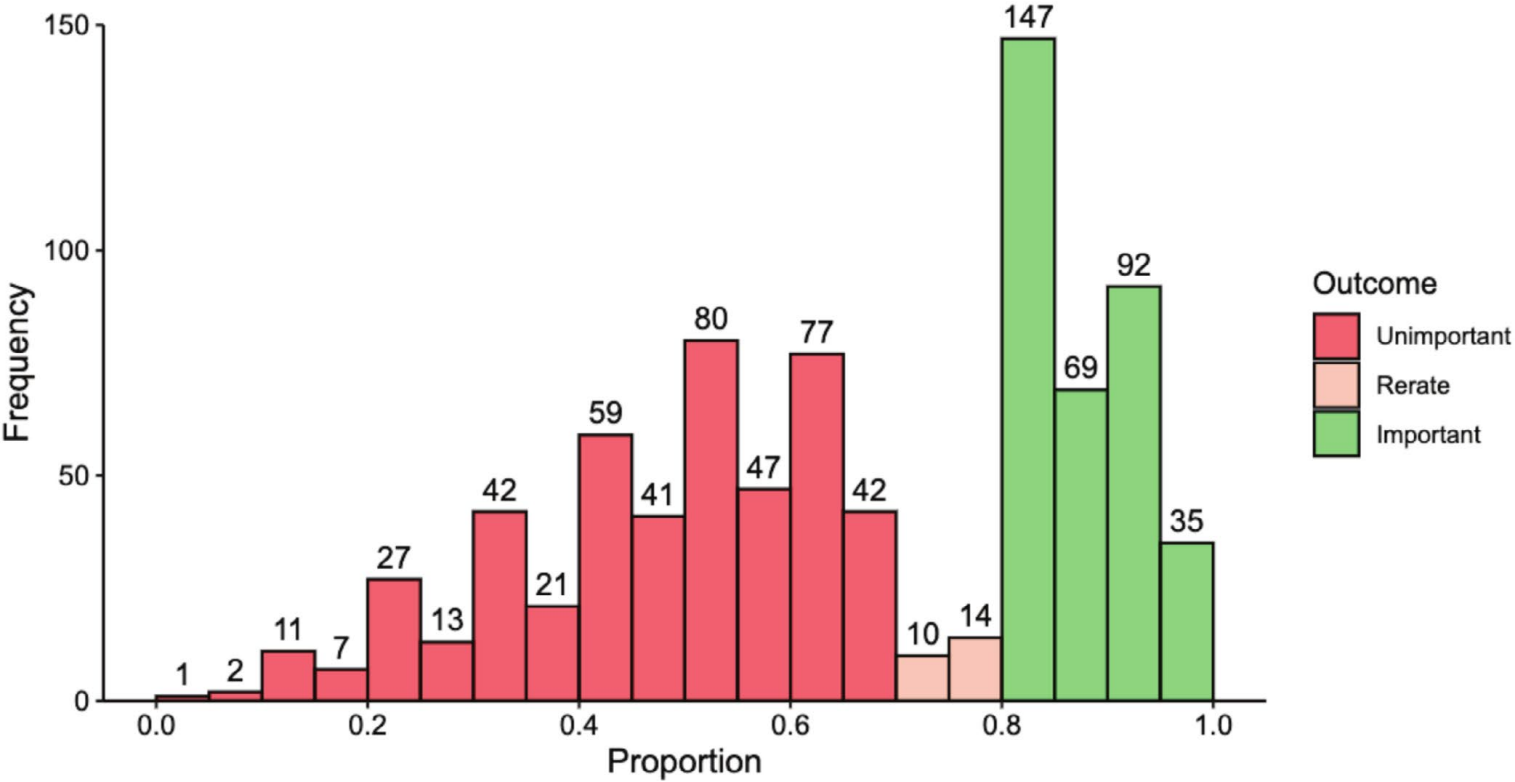
Proportion of statements rated as essential or important across the study. These values reflect the final time a statement was rated (as statements could be re‐rated). Columns are binned in 5% increments (for ease of interpretation).

**FIGURE 4:**
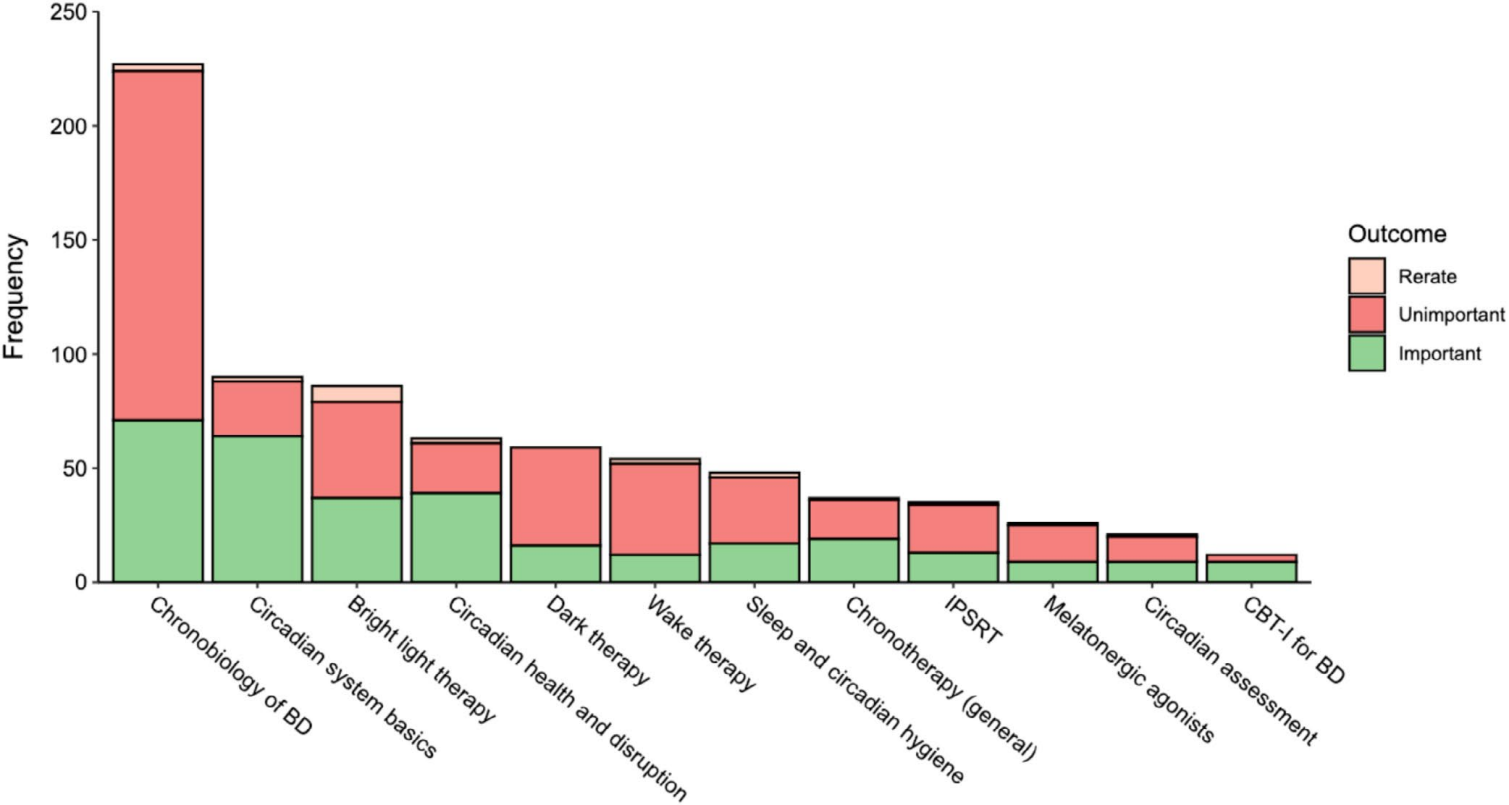
Patterns of ratings of statements categorised within core constructs. The 12 constructs were created by the investigators to ease cognitive load when rating, and the columns are ordered according to the number of statements within each construct.

Across three rounds, the experts reached consensus (≥ 80% agreement) on 342 statements that are essential for clinicians supporting people with BD to know about the chronobiology and chronotherapeutics of BD. Visualisations of the distributions of ratings for all statements are available on the *Open Science Framework* (https://osf.io/agk9y). These consensus statements spanned four key themes (discussed in detail below): (1) basic circadian science; (2) circadian health and disruption; (3) chronobiology of BD; and (4) chronotherapies. Figure 2 illustrates the flow of statements across the three rounds of the study. Figure 3 provides a summary of the distribution of ratings across all the statements examined. Figure 4 shows the distribution of the statements across the 12 core constructs.

Given constraints on space, we summarise a selection of 60 consensus statements in Table 2 (chosen to have representation of five statements from each of the 12 core constructs). These represent only one‐sixth (17.5%) of the consensus statements. We encourage readers to explore the total set, available on the *Open Science Framework* (https://osf.io/agk9y). For ease of comprehension, we also provide links to access the 342 consensus statements organised according to the 12 core constructs (Table 3).

**TABLE 2:** Summary of 60 consensus statements, selected to have representation of five statements from each of the 12 core constructs.

	%
Core construct 1: Basics of the mammalian circadian system	
The suprachiasmatic nucleus (SCN) has an intrinsic rhythm which is constantly entrained by external stimuli	100
Some people's sleep–wake rhythm starts later (e.g., night owls) than others (e.g., early‐birds or morning larks)	100
The light/dark cycle is the strongest zeitgeber in humans	100
Melatonin synthesis is inhibited by exposure to light	100
Melatonin is a chemical signal of darkness	97
Core construct 2: Circadian health and disruption	
Increasing morning light exposure can shift the biological clock to an earlier phase and lessen social jetlag	100
Decreasing evening light exposure can shift the biological clock to an earlier phase and lessen social jetlag	100
Irregularity of the sleep/wake cycle is associated with greater mood instability	100
Circadian rhythm disorders can be caused by internal factors (e.g., genetic variation) or external factors (e.g., shift work, jet lag)	97
The relationships between the sleep–wake cycle and mental health are bi‐directional	93
Core construct 3: Assessing circadian rhythms in clinical practice	
Discussion about chronotype should be a regular part of psychoeducation, and can introduce people with BD to the area of circadian rhythms and its relevance to mood disorders	90
Melatonin rhythms are one of the most direct and robust indicators of suprachiasmatic nucleus (SCN) timing	87
Actigraphy can assess the timing and amplitude of the 24‐h rest‐activity rhythm	87
When there are significant differences between weekday and weekend rest‐activity behaviour, this is called ‘social jetlag’	87
Some output rhythms are direct or pure expressions of suprachiasmatic nucleus (SCN) activity (e.g., melatonin secretion, core body temperature, cortisol), while others are products of circadian and social‐behavioural processes (e.g., rest‐activity rhythm)	87
Core construct 4: Chronobiology of BD	
Symptoms of BD can interfere with the ability to sleep	100
Seasonal changes, like changes in day length, are a common trigger for mood changes and mood episodes in BD	100
Situations involving rapid changes in timing of the ambient light/dark cycle (e.g., international travel, shift work, stimulants, childbirth) can trigger mood symptoms and episodes in BD.	100
A malfunctioning body clock may underlie the mood cycles in BD	97
Poor sleep can worsen symptoms of BD	97
Core construct 5: Chronotherapy: General concepts	
Chronotherapies are techniques that affect circadian rhythms and the central circadian clock	97
Chronotherapies for affective disorders are treatments that seek to alter mood‐states by affecting circadian rhythms and the central circadian clock	
Chronotherapies use manipulation of sleep, wake, and light	97
The 5 major modalities of affective chronotherapies are: bright light therapy; dark therapy; melatonin and melatonergic agonists; treatments using sleep deprivation or wake therapy; interpersonal and social rhythm therapy; and cognitive behavioural therapy for insomnia adapted for BD	97
The integrated treatment approach to BD should include attention to the 24 h light/dark cycle as well as a focus on the sleep–wake cycle	97
Core construct 6: Sleep and circadian hygiene	
People with BD should get out of bed at the same time each day	97
To sleep well, people with BD should avoid stimulants (e.g., caffeine, nicotine, chocolate) late in the day	97
For people with BD, it may be helpful to change their attitude to sleep (e.g., welcome it as a contributor to wellbeing and not wasted time)	93
People with BD should turn off screens, phones, and other devices 1–2 h before bedtime	93
Regular routines and sleep schedules may lengthen the time between manic or hypomanic episodes	93
Core construct 7: Bright light therapy	
Timing of exposure affects the outcome and tolerability of bright light therapy	97
Distance from the light device affects the outcome and tolerability of bright light therapy	97
When using bright light therapy, provided that a person with BD is treated with appropriate mood‐stabilising medications and has no additional risk factors, the risk of switching into hypomania or mania appears to be relatively rare	97
Bright light therapy has strong evidence as an antidepressant for bipolar depression	93
Bright light therapy is well‐tolerated in BD	93
Core construct 8: Wake therapy	
There is a slight risk of switching into mania for a person with BD receiving wake therapy	90
Sleep deprivation therapies work rapidly	87
By themselves, sleep deprivation therapies lead to only transient improvements in mood	87
Therapeutic sleep deprivation is typically combined with medication or other chronotherapies, particularly bright light therapy and sleep phase advance	87
When used for acute bipolar depression, therapeutic sleep deprivation should always be combined with mood stabilising medication	83
Core construct 9: Dark therapy	
Modern dark therapy involves wearing special orange tinted glasses which block the blue wavelength from artificial and natural light	100
“Virtual darkness” can be created by wearing blue light blocking glasses	97
When not in a manic episode, blue‐blocking glasses can be worn at a regular time, about 2 h before bed, in order to promote regular endogenous melatonin‐production and better sleep.	93
Blue‐blocking glasses may be useful for treating mania in BD	90
A person with BD can darken their bedroom using black‐out curtains	90
Core construct 10: Interpersonal and Social Rhythms Therapy	
In Interpersonal and Social Rhythm Therapy (IPSRT), people learn to identify and manage events that destabilise their daily routines	100
In IPSRT, people learn to track the regularity of 5 activities: (1) time out of bed; (2) first contact with another person; (3) time starting formal commitments; (4) dinner; and (5) bed‐time	97
In IPSRT, the Social Rhythm Metric is used to track and improve the regularity of social activities	90
IPSRT improves functioning in people with BD	90
IPSRT works for BD because people with BD are vulnerable to dysregulation of daily rhythms	83
Core construct 11: Melatonin and melatonergic agonists	
There is insufficient evidence to endorse or reject melatonin for the acute phase of bipolar depression	87
There is insufficient evidence to endorse or reject melatonergic agonists for the acute phase of bipolar depression	87
There is insufficient evidence to endorse or reject melatonergic agonists for prophylaxis of bipolar depression	83
Taking melatonin an hour or so before the desired bedtime sends an entraining signal to the circadian clock	83
Melatonin quality is variable, suggesting the need for use only of approved products	83
Core construct 12: Cognitive behaviour therapy for insomnia adapted for BD	
Cognitive behaviour therapy for insomnia (CBT‐I) addresses thoughts and beliefs that interfere with sleep and teaches sleep hygiene practices	97
CBT‐I for BD (CBT‐I‐BD) aims to improve mood, sleep, and functioning in people with BD	97
CBT‐I may improve insomnia in BD and other mood disorders	90
CBT‐I‐BD produces benefits for sleep after acute treatment among people with inter‐episode BD and comorbid insomnia	87
CBT‐I‐BD produces benefits for mood episode relapse after acute treatment among people with inter‐episode BD and comorbid insomnia	87

*Note:* % indicates the proportion of experts rating statement as Essential/Important.

**TABLE 3:** Consensus statements organised according to 12 core constructs.

Core constructs	Access the statements
1. Basics of the mammalian circadian system	https://osf.io/ktq2x
2. Circadian health and disruption	https://osf.io/pnuxg
3. Assessing circadian rhythms in clinical practice	https://osf.io/qg3pc
4. The chronobiology of BD	https://osf.io/d4rmq
5. Chronotherapy: General concepts	https://osf.io/pgj4w
6. Sleep and circadian hygiene	https://osf.io/f3acd
7. Bright light therapy	https://osf.io/mz7kd
8. Wake therapy	https://osf.io/kc7nx
9. Dark therapy	https://osf.io/g4qjv
10. Interpersonal and Social Rhythms Therapy	https://osf.io/mx8ub
11. Melatonin and melatonergic agonists	https://osf.io/ctfkx
12. CBT for insomnia adapted for BD	https://osf.io/6eqwp

#### 3.1.2 Consensus Theme 1: Basic Circadian Science

It is essential that clinicians know about basic aspects of circadian science. Consensus was reached on 70 statements in this theme. All experts agreed that clinicians should know that the pacemaker of the circadian system (the *suprachiasmatic nucleus* [SCN]) has an intrinsic rhythm that is constantly entrained by external stimuli (e.g., light); that some people's sleep–wake cycle starts later (*night owls*) than others (*morning larks*); and that melatonin synthesis is inhibited by exposure to light (all but one expert thought that clinicians should know that melatonin is the ‘chemical signal of darkness’—this is in contrast to the popular idea that melatonin is a ‘sleep hormone’).

Other consensus statements included topics spanning *definitions* (e.g., phase, period) and *types* of circadian rhythms (e.g., core body temperature, cortisol); that circadian rhythms are widespread throughout the body and are found in almost all cells; that the circadian system regulates many processes relevant to BD (e.g., mood, cognition, hormone secretion, gene expression); that circadian rhythms can shift earlier or later relative to clock time; that zeitgebers (German for ‘time‐givers’) entrain the circadian system (with zeitgebers including the timing of light, eating, work, exercise, social activity); that melatonin is a key circadian signal; that there are direct effects of light on brain areas relevant to mood and cognition; that the circadian system is most sensitive to short‐wavelength ‘blue’ light (460‐480 nm); and that ‘blue’ light promotes wakefulness and suppresses melatonin.

Consensus in this theme may reflect the idea that if clinicians are aware of the ‘what’, ‘how’, and ‘why’ of the circadian system, then they may be more motivated to engage with circadian principles and to correctly employ the chronotherapies in Theme 4.

#### 3.1.3 Consensus Theme 2: Circadian Health and Disruption

It is essential that clinicians know about the causes of circadian disruption and consequences for health and wellbeing. Consensus was reached on 42 statements related to this theme. All experts agreed that clinicians should know that increasing morning light exposure and decreasing evening light exposure can shift the timing of the circadian clock earlier, and that irregularity of the sleep–wake cycle is associated with greater mood instability. Other consensus statements were that circadian rhythm disorders are multifactorial and can be caused by internal (e.g., genetic variation) and external factors (e.g., shift work, jet lag); that the relationships between the sleep–wake cycle and mental health are bi‐directional; that circadian disruption is associated with risk of mood instability and mood disorders; that evening chronotype is associated with higher risk of mood disorders; and that knowing one's chronotype can help them understand their internal clock and how best to synchronise it.

Consensus within this theme may reflect two ideas: (1) clinicians should know about circadian disruptors and how people with BD can minimise disruption by changing their behaviour and environments; and (2) clinicians should be equipped with principles that can help them predict the illness course of their patients (e.g., seasonal patterns).

#### 3.1.4 Consensus Theme 3: Chronobiology of BD

It is essential that clinicians know about key aspects of the chronobiology of BD. Consensus was obtained on 73 statements about the chronobiology of BD and 11 statements about assessment of circadian rhythms in clinical practice. Consensus statements included the role of circadian processes in the aetiology and course of BD; that the phenomenology of BD encompasses processes regulated by circadian rhythms (e.g., mood, appetite, energy); that sleep disturbance is a prognostic signal in the prodrome of mania; that sleep disturbance often endures inter‐episodically in BD; that people with BD may have altered sensitivity to zeitgebers (i.e., time cues) like light; that there are bidirectional relationships among symptoms of BD and sleep, the sleep–wake cycle, and circadian rhythms; that factors that disrupt the sleep–wake cycle (e.g., international travel, shift work, childbirth) affect mood changes in BD; that there is meaningful inter‐individual variability in sleep and circadian features in BD; that sleep disruption is associated with serious clinical outcomes (e.g., suicidality); that people with BD may be hyper‐sensitive to the circadian‐disrupting effects of artificial light at night; that stabilisation of circadian rhythms is a therapeutic mechanism in BD; that lithium can phase shift circadian rhythms; and that stabilisation of circadian rhythms may be an essential mechanism of certain therapies (e.g., lithium).

Consensus within this theme may reflect the notion that the circadian system is implicated in many aspects of BD and that improving circadian rhythmicity is therefore a logical therapeutic strategy.

#### 3.1.5 Consensus Theme 4: Chronotherapies

Finally, it is essential that clinicians know about the six major affective chronotherapies, including evidence in support of them and information about their procedures (e.g., dose, timing), outcomes, side effects, and contraindications. Comments from the experts on specific statements emphasised a need for more rigorous clinical trials, and post‐study correspondence highlighted that this consensus paper would lay the foundation for future collaborative work in clinical trials.

##### 3.1.5.1 Bright Light Therapy

This chronotherapy had the most consensus statements (*n* = 60). In addition to the statements in Table 2, clinicians should know about key parameters that affect the outcome and tolerability of BLT (e.g., intensity of light [lux], wavelength, device angle, exposure duration); Bright light therapy (BLT) should provide 7000–10,000 lx at a distance of 30–38 cm (12–15 in.) for 30–120 min per day (depending on light output); that for the depressed phase of BD, midday administration may be effective, whereas nighttime administration is not recommended; adverse effects appear to be mild; that a trained clinician can recommend which type of device can be used; and that BLT should be used under clinical supervision.

##### 3.1.5.2 Wake Therapy

This chronotherapy had 12 consensus statements. In addition to those in Table 2, clinicians should know that wake therapy should only be used under clinical supervision; that adding BLT to wake therapy can prolong its therapeutic effect; that wake therapy is used only during the depressed phase of BD because it can worsen or trigger manic symptoms; and that wake therapy may not be suitable for people with comorbid epilepsy (as it may induce seizures).

##### 3.1.5.3 Dark Therapy

Most of the 20 consensus statements were related to modern dark therapy (blue‐blocking glasses [BBGs]). Clinicians should know that dark therapy is not a substitute for medication in BD; that BBGs should block 90% of blue light; that BBGs can improve sleep efficiency and lead to a rapid resolution of mania in hospitalised people; that while manic, people with BD should wear BBGs from ~6 pm until getting into bed and turning off lights, and then again in the morning if they are awake, until 8 am; and that people with BD can reduce their environmental exposure to light at night using an eye mask or black‐out curtains.

##### 3.1.5.4 Interpersonal and Social Rhythms Therapy

There were 13 consensus statements about Interpersonal and Social Rhythms Therapy (IPSRT). In addition to statements in Table 2, clinicians should know that IPSRT and CBT can be combined together and also combined with mood stabilisers and antipsychotic medications; that IPSRT appears feasible to deliver in people with BD and to youth at risk of BD; that IPSRT is a promising adjunctive for adolescents with BD; that Social Rhythm Therapy can improve mood in youth with BD; and that IPSRT reduces depressive symptoms in people with BD.

##### 3.1.5.5 Melatonin and Melatonergic Agonists

Most of the 10 consensus statements indicated that there is insufficient evidence to endorse or reject melatonin or melatonergic agonists for the acute phases or prophylaxis of bipolar depression and mania in BD.

##### 3.1.5.6 Cognitive Behaviour Therapy for Insomnia Adapted for BD

There were nine consensus statements related to this chronotherapy. Clinicians should know about the structure and aims of CBT‐I and CBT‐I‐BD; that Cognitive Behaviour Therapy for Insomnia Adapted for BD (CBT‐I‐BD) can improve sleep and reduce relapse after acute treatment in people with inter‐episode BD and comorbid insomnia; and that a package that combines CBT‐I, psychoeducation, and light–dark cycle management can improve sleep and mental health in inpatients with BD.

## 4 Discussion

This ISBD Chronobiology and Chronotherapy Task Force study identified a core knowledge base about chronobiology and chronotherapeutics for clinicians, which is intended to support and optimise their care of people with BD. Using a Delphi process with 30 experts from 15 countries (Figure 1), ~750 statements were distilled to a final set of 342 consensus statements. This process unpacks the chronobiological model of BD into practices and principles that will enable this paradigm to be more effectively employed in the understanding and treatment of BD.

### 4.1 Dissemination

Dissemination and implementation science investigates how information is distributed and assimilated by clinicians. It seeks to determine the ‘how, what, when, where, and to whom’ findings are best incorporated and utilised in clinical practice. To determine the optimal strategies for the translation of this consensus information, we need to consider several challenges including the content and size of the study's final set of statements. To start with, the 342 statements deemed as essential or important by our experts could form a large and ambitious educational package. We did not impose an a priori limit on its scope, allowing experts to choose and rank statements without concern for the ultimate set size of the set. Given its large size, how can this information be optimally organised, and through what channels can it be distributed to most effectively educate clinicians?

First, most of the experts believe that dissemination of this chronobiological and chronotherapeutic model of BD is best supported through its incorporation into CPGs and training curricula (see Figure S1). As one of the now‐foundational models of the pathophysiology of BD, meaningful dissemination of the science requires inclusion of this area in CPGs and graduate and postgraduate clinical education. Extrapolating from the sleep medicine literature [34, 40, 56, 57] and our anecdotal experience, we believe there is a paucity of information about chronobiology in clinical training programs and professional guidelines for the treatment of BD. The knowledge base identified here may help address this. The breakdown of these consensus statements into four major themes (i.e., ‘basic circadian science’; ‘circadian health and disruption’; ‘chronobiology of BD’; and ‘chronotherapies’) lends itself to a multi‐session or multi‐module educational series, whether through a short course of classes, webinars, or Continuing Medical Education materials.

This information could also be refined and tailored to specific settings. For example, clinicians with different training requirements may benefit from learning different levels of this knowledge base. While psychiatrists might ideally employ the full breadth of these principles and practices, psychologists, nurses, and primary care physicians may require a condensed version or a focus on ‘general circadian psychoeducation’ (Table S1 provides a heuristic framework for how information might be tailored to clinician types). Educational programs could be customised to specific clinical situations such as the initial assessment and formulation, management of acute episodes, maintenance therapy, and treatment and prevention of specific chronobiological challenges (e.g., seasonal change, shift work). In Box 2, we provide one example of how a sample of the consensus items could be applied to guidance about the management of inter‐episode mood instability.

BOX 2 **An example of how consensus statements can be applied to education about the management of inter‐episode mood instability.**

**Table:** 

Circadian principles	Circadian disruption is associated with greater mood instability Stabilisation of circadian rhythms is therapeutic in BD People with BD may need stronger time cues (zeitgebers) to have regular circadian rhythms Strictly regulating the sleep‐wake schedule can improve extreme mood cycles in BD
General circadian psychoeducation	*Sleep–wake regulation*: Encourage people with BD to: ○ Get out of bed at the same time each day ○ Ensure their bedroom is cool, quiet, dark, and free of distractions (e.g., TV, screens) ○ Get at least 7 hours of sleep at night (for most individuals) ○ Avoid stimulants (e.g., caffeine, nicotine, chocolate) late in the day ○ Engage in regular physical activity *Managing night‐time light exposure*: Encourage people with BD to: ○ Avoid exposing themselves to light‐sources containing blue light during the main rest interval (night) ○ Turn off screens, phones, and other devices 1‐2 hours before bedtime ○ Reduce night‐time light exposure in the bedroom with an eye mask *Managing day‐time light exposure*: Encourage people with BD to: ○ Spend time in daylight, as indoor living dampens the natural signals that set the biological clock
Advanced practice (specialists)	*Clinicians can consider prescribing*: Mid‐day bright light therapy ○ Which may be effective in reducing depressive symptoms ○ Appears to have mild adverse effects. It is not suitable for people with certain types of severe eye disease. ○ When used, the device should: Provide 7000–10,000 lux at a distance of 12–15 inches/30–38 cm from the eyes (with the light source mounted slightly above eye level) Be used for 30 minutes to 2 hours (depending on the light intensity and clinical factors) Have a protective UV filter Blue‐blocking glasses (i.e., modern dark therapy) ○ This involves prescribing special orange tinted glasses which block the blue wavelength from artificial and natural light, and which effectively blocks the activating and melatonin‐suppressing effects of light. The best evidence for blue light reduction is for mania, mixed states, and rapid cycling ○ When not in a manic episode, blue‐blocking glasses can be worn at a regular time, about 2 hours before bed, in order to promote regular endogenous melatonin‐production and better sleep Interpersonal and Social Rhythm Therapy (IPSRT) ○ To educate people to identify and manage events that destabilise their routines ○ To support people to track the regularity of 5 activities: (1) time out of bed; (2) first contact with another person; (3) time starting formal commitments; (4) dinner; and (5) bed‐time ○ The Social Rhythm Metric can be used to track and improve the regularity of social activities Cognitive behavioural therapy for insomnia (CBT‐I) ○ Addresses thoughts and beliefs that interfere with sleep and teaches sleep hygiene practices ○ CBT for BD (CBTI‐BD) is a modification with added elements of motivational interviewing and IPSRT ○ CBTI‐BD produces benefits for mood episode relapse after acute treatment among people with inter‐episode BD and comorbid insomnia

*Note:* Some statements were rephrased slightly to minimise repetition.

Another question bearing on effective dissemination is whether this information is best organised as a stand‐alone chronobiological model of care, or whether these practices and principles would be better included in existing, evidence‐based therapies such as psychoeducation or IPSRT. Recent proposals of a ‘circadian type’ of depression might support the use of pathophysiology‐targeted, stand‐alone interventions [19]. However, we believe that many of the consensus statements obtained are so important and widely relevant that they should be part of the standard of care for all clinicians working in mood disorders (Table S1). This would include, for example, the need to manage the timing and degree of light and dark exposure. Other items, such as how to implement the less common and complex chronotherapies (e.g., wake therapy), might be reserved for specialised classes or educational forums (Box 2).

Finally, there have been major innovations in the estimation of circadian parameters in real‐world settings via application of mathematical models to wearables [58, 59]. The combination of improved subject knowledge among clinicians, real‐time continuous measurement of behaviour [23, 60, 61], and models that can estimate circadian rhythms and deliver this information digitally may offer new opportunities to personalise and scale chronotherapeutic management strategies for BD.

### 4.2 Limitations

Several methodological limitations are worth mentioning. First, our recruitment strategy—which leveraged the mailing lists of several major professional organisations in this topic area with the addition of snowball sampling—unfortunately failed to attract three kinds of experts: (a) residents of the African continent and two of the world's most populated countries (India, China); (b) residents of several countries facing unique circadian challenges (e.g., polar nights) caused by their latitude (e.g., Finland, Iceland); and (c) non‐psychiatrist health professionals that play a role in the management of BD (e.g., psychologists, general practitioners, mental health nurses). This may bias the generalisability of the consensus information and may have prevented rating of information specific to these contexts. Second, given the diversity and complexity of this area, a large number of statements were rated in Round 1. This was burdensome and, in some cases, caused attrition in Round 1, though minimal in degree. Third, given that most experts are members of a task force dedicated to the chronobiology and chronotherapy of BD, it is possible that the consensus information is biased toward greater inclusiveness of information, as compared to what might have been derived by generalists or specialists in other areas of BD. Finally, only two people with lived experience of BD were involved in the conduct of the study. We emphasise that these individuals are *lived experience researchers* [53] who have specialised knowledge and expertise in the typically disconnected domains of research on mood disorders and personal lived experience of a mood disorder; this coupling is a major strength of their involvement in this study. However, we acknowledge that inclusion of a broader group of people from the community who have lived experience of BD may have captured conflicting experiential knowledge, and allowed for novel insights about the value and limitations of the chronobiological model of BD. We note that future offshoots of our work, such as self‐management of BD using chronobiological principles (wherein people with lived experience are the end‐users), would clearly lend itself to complete involvement of lived experience in the formal rating process of the Delphi study.

## 5 Conclusion

We obtained an expert consensus about concepts, principles, and practices related to the chronobiology and chronotherapy of BD, which experts think are essential for clinicians to know to optimise their care of people with BD. Our next step will be to organise and disseminate this information, similar to the Society for Research on Biological Rhythms ‘Circadian Medicine Course’ [41] but with a BD focus. We expect that the uptake of this consensus information will improve the outcomes of people with BD, especially if the findings are incorporated in CPGs and clinical training programs [25]. We also invite readers to use the full consensus body of information in their own educational and training programs and curricula (https://osf.io/agk9y).

## Author Contributions

**Jacob J. Crouse:** data curation, formal analysis, funding acquisition, investigation, methodology, project administration, software, visualization, writing – original draft, writing – review and editing. **Victoria Loblay:** data curation, formal analysis, investigation, methodology, project administration, writing – review and editing. **Anthony Jorm:** methodology, writing – review and editing. **Timothy R. Wong:** data curation, formal analysis, software, visualization, writing – review and editing. **Zsofi de Haan:** writing – review and editing. **Carla Gorban:** writing – review and editing. **Mirim Shin:** writing – review and editing. **Emiliana Tonini:** writing – review and editing. **Chris Aiken:** writing – review and editing. **Lauren B. Alloy:** writing – review and editing. **Kürşat Altinbaş:** writing – review and editing. **Serge Beaulieu:** writing – review and editing. **Joanne S. Carpenter:** writing – review and editing. **Bruno Etain:** writing – review and editing. **Yuichi Esaki:** writing – review and editing. **Jess G. Fiedorowicz:** writing – review and editing. **Corrado Garbazza:** writing – review and editing. **Pierre A. Geoffroy:** writing – review and editing. **Bartholomeus C. M. Haarman:** writing – review and editing. **Tone E. G. Henriksen:** writing – review and editing. **Maria Paz Hidalgo:** writing – review and editing. **Maree L. Inder:** writing – review and editing. **Raymond W. Lam:** writing – review and editing. **Helle Ø. Madsen:** writing – review and editing. **Colleen A. McClung:** writing – review and editing. **Gunnar Morken:** writing – review and editing. **Laura Palagini:** writing – review and editing. **James Phelps:** writing – review and editing. **Richard J. Porter:** writing – review and editing. **Aswin Ratheesh:** writing – review and editing. **Rixt F. Riemersma‐Van der Lek:** writing – review and editing. **Janusz K. Rybakowski:** writing – review and editing. **Erika F. H. Saunders:** writing – review and editing. **Peter F. J. Schulte:** writing – review and editing. **Daniel J. Smith:** writing – review and editing. **Holly A. Swartz:** writing – review and editing. **Bryan K. Tolliver:** writing – review and editing. **Anna Wirz‐Justice:** writing – review and editing. **Ian B. Hickie:** writing – review and editing. **John F. Gottlieb:** conceptualization, data curation, formal analysis, investigation, methodology, project administration, writing – original draft, writing – review and editing.

## Funding

NHMRC Emerging Leadership Fellowship (2008196) awarded to J.J.C.

## Conflicts of Interest

J.J.C. declares funding from the National Health and Medical Research Council and Wellcome Trust. S.B. declares peer‐reviewed research funding from CIHR; research support, KT, contract, investigator‐initiated trial from Diamentis and Otsuka; consultant‐advisory board membership for Abbvie, Boheringer, Janssen‐Ortho, Lundbeck, Otsuka, Sunovion, and Takeda; Speaker Bureau for Abbvie, Janssen‐Ortho, Lundbeck, Otsuka, Sunovion; and board member of Relief (myRelief.ca) (pro bono). L.B.A. discloses funding from NIMH R01 126,911. B.E. discloses funding from Agence Nationale de Recherche, Fondation Fondamental, and Sanofi. P.A.G. discloses payment or honoraria from Arrow, Biocodex, Di&Care, Idorsia, Janssen‐Cilag, Jazz pharmaceuticals, Myndblue, and Pharmanovia; B.C.M.H. discloses a ZonMw Postdoc Fellowship (Netherlands Organisation for Health Research and Development; grant number 636320010); LivaNova Investigator Initiated Research grant; Health Holland Public Private Partnership allowance; and payment or honoraria from WAD Cursus (https://www.wadcursus.nl). T.E.G.H. discloses payment for lectures on chronotherapies fort the specialisation program for psychiatrists, Forde Health Trust, Norway (2024) and Stavanger Health Trust, Norway (2023); is a member of the Scientific advisory board for Good Light Group; and donation of blue blocking glasses from Melamedic.dk and Somnoblue for use in research; R.W.L. discloses grants/contracts from BC Leading Edge Endowment Fund, Brain Canada, Canadian Institutes of Health Research, Canadian Network for Mood and Anxiety Treatments, Grand Challenges Canada, Healthy Minds Canada, Janssen, Michael Smith Foundation for Health Research, MITACS, Ontario Brain Institute, Unity Health, Vancouver Coastal Health Research Institute, VGH‐UBCH Foundation; royalties or licences from Cambridge University Press, Oxford University Press; payment or honoraria from Carnot, Canadian Medical Protective Association, Lundbeck, Neurotorium, Otsuka, Shanghai Mental Health Center; participation on a DSMB or Advisory Board for AbbVie, Bausch, and Otsuka; and leadership or fiduciary role for Asia‐Pacific Economic Cooperation, Canadian Network for Mood and Anxiety Treatments, CB Solutions, and Genome BC. L.P. discloses consulting fees, payment or honoraria, and participation on a DSMB or advisory board for Bruno, Idorsia, Italfarmaco, Fidia, Neopharmed Gentili, Pfozer, Sanofi, Pharmanutra, and Viatris. J.P. discloses royalties or licences from McGraw‐Hill and W.W. Norton & Co. R.J.P. discloses grants from Wellcome Trust, Health Research Council of New Zealand, Canterbury Medical Research Foundation, and Lotteries Health NZ; support for attending meetings and/or travel from Lundbeck Australia and Servier Australia; and provision of software from SBT Pro. E.F.H.S. discloses support for attendings meetings/travel as a board member of International Society for Bipolar Disorders (ISBD); and leadership or fiduciary roles for ISBD, American Society for Clinical Psychopharmacology, American Association of Chairs of Departments of Psychiatry, and National Network of Depression Centers. E.F.H.S. discloses grants or contracts from National Institute of Mental Health, National Science Foundation, and American Foundation for Suicide Prevention; consulting fees from Mediflix, Postgrad Physician Press, Intracellular Therapies; payment or honoraria from WebMD/Medscape and Clinical Education Alliance; support for attending meetings and/or travel from ISBD; participation on a DSMB or advisory board for grants R01 MH125155 and R01 MH109662; leadership or fiduciary roles for ISBD, American Society for Clinical Psychopharmacology, International Society of Interpersonal Psychotherapy, and Depression and Bipolar Support Alliance; and other financial or non‐financial interests for American Journal of Psychotherapy (Editor‐in‐Chief), UpToDate/Wolters Kluwer (royalties), and American Psychiatric Association (royalties). I.B.H. discloses grants from National Health and Medical Research Council; consulting fees as an advisory board member for Janssen Cilag; payment or honoraria from Janssen Cilag; leadership or fiduciary role for the Australian Department of Health; and Chief Scientific Advisor to, and a 3.2% equity shareholder in, InnoWell Pty Ltd. The other authors declare no conflicts of interest.

## Supporting information


**Figure S1:** Recommended avenues for dissemination of consensus information.
**Table S1:** A heuristic of ‘who’ the consensus statements might be most ‘essential’ for.

## Data Availability

The data that support the findings of this study are available on request from the corresponding author. The data are not publicly available due to privacy or ethical restrictions.
