# Supplementary material for: Essential Information About Chronobiology and Chronotherapy for the Optimal Care of People With Bipolar Disorders: An Expert Consensus
Source: Bipolar Disord. 2026 Jul 10;28(5):e70141. doi: 10.1111/bdi.70141 (PMC13352581; doi:10.1111/bdi.70141)
Supplement: Supplementary file 1 — Figure S1: Recommended avenues for dissemination of consensus information. Table S1: A heuristic of ‘who’ the consensus statements might be most ‘essential’ for. [file BDI-28-0-s001.docx]

**SUPPLEMENTARY MATERIAL**

**Table of contents**

1. Grey literature search
2. Figure S1. Recommended avenues for dissemination of consensus information.
3. Table S1. A heuristic of “who” the consensus statements might be most “essential” for.

**Grey literature search**

Searches were conducted on May 5, 2023, using Google in the US, UK, and Australia. Ten strings of search terms were used which captured key elements related to bipolar disorder, including (but not limited to): “mania”, “bipolar depression”, “circadian”, “chronobiology”, “chronotherapy”, “sleep-wake”, “chronotype”, “social jet lag”, “light therapy”, and “social rhythm therapy”. The 10 strings of terms used are reported in the Supplementary Materials. For each of these 10 strings, we extracted links from the first 10 pages on Google (n=100), and conducted this separately for the US, UK, and Australian sites. A total of 3000 records were extracted. After removal of duplicates, 1596 unique records were manually screened. 470 records were excluded for being a grant, preprint, refereed publication, or clinical trial registration. This left 1126 records that were available for grey literature synthesis. The most common forms of text were blog posts, explainers, news articles, and press releases. J.J.C. read each record and extracted items related to the topic area.

**Figure S1. Recommended avenues for dissemination of consensus information.**

**
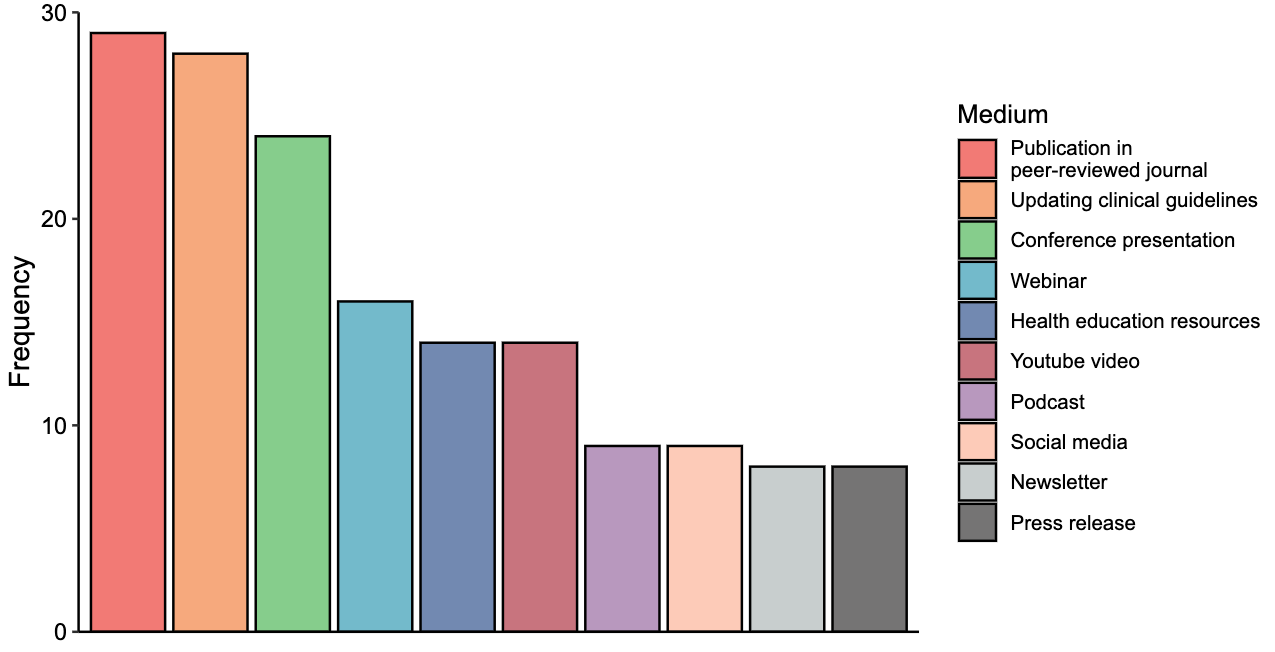
**

**Table S1. A heuristic of “who” the consensus statements might be most “essential” for.**

| **Green** = most essential  **Yellow** = less essential  **Red** = least essential | Psychiatrist | Psychologist | Primary care physician | Mental health nurse | Social worker | Case manager | Occupational therapist | Family therapist | Peer worker |
| --- | --- | --- | --- | --- | --- | --- | --- | --- | --- |
| **Core constructs** | | | | | | | | | |
| 1. Basics of the mammalian circadian system |  |  |  |  |  |  |  |  |  |
| 2. Circadian health and disruption |  |  |  |  |  |  |  |  |  |
| 3. Assessing circadian rhythms in clinical practice |  |  |  |  |  |  |  |  |  |
| 4. The chronobiology of BD |  |  |  |  |  |  |  |  |  |
| 5. Chronotherapy: General concepts |  |  |  |  |  |  |  |  |  |
| 6. Sleep and circadian hygiene |  |  |  |  |  |  |  |  |  |
| 7. Bright light therapy |  |  |  |  |  |  |  |  |  |
| 8. Wake therapy |  |  |  |  |  |  |  |  |  |
| 9. Dark therapy |  |  |  |  |  |  |  |  |  |
| 10. Interpersonal and Social Rhythm Therapy |  |  |  |  |  |  |  |  |  |
| 11. Melatonin and melatonergic agonists |  |  |  |  |  |  |  |  |  |
| 12. CBT for insomnia adapted for BD |  |  |  |  |  |  |  |  |  |
